# Automated quantification of baseline imaging PET metrics on FDG PET/CT images of pediatric Hodgkin lymphoma patients

**DOI:** 10.1186/s40658-020-00346-3

**Published:** 2020-12-14

**Authors:** Amy J. Weisman, Jihyun Kim, Inki Lee, Kathleen M. McCarten, Sandy Kessel, Cindy L. Schwartz, Kara M. Kelly, Robert Jeraj, Steve Y. Cho, Tyler J. Bradshaw

**Affiliations:** 1 Department of Medical Physics, University of Wisconsin-Madison, Madison, WI USA; 2 Department of Radiology, University of Wisconsin-Madison, Madison, WI USA; 3 Department of Nuclear Medicine Korea Cancer Centre Hospital, Korea Institute of Radiological and Medical Sciences, Seoul, Korea; 4 IROC-Rhode Island, Lincoln, RI USA; 5 Medical College of Wisconsin, Milwaukee, WI USA; 6 Department of Pediatrics, Roswell Park Comprehensive Cancer Center, University at Buffalo Jacobs School of Medicine and Biomedical Sciences, Buffalo, NY USA; 7 University of Wisconsin Carbone Comprehensive Cancer Center, Madison, WI USA

**Keywords:** Pediatric lymphoma, Convolutional neural networks, Imaging biomarkers, PET

## Abstract

**Purpose:**

For pediatric lymphoma, quantitative FDG PET/CT imaging features such as metabolic tumor volume (MTV) are important for prognosis and risk stratification strategies. However, feature extraction is difficult and time-consuming in cases of high disease burden. The purpose of this study was to fully automate the measurement of PET imaging features in PET/CT images of pediatric lymphoma.

**Methods:**

^18^F-FDG PET/CT baseline images of 100 pediatric Hodgkin lymphoma patients were retrospectively analyzed. Two nuclear medicine physicians identified and segmented FDG avid disease using PET thresholding methods. Both PET and CT images were used as inputs to a three-dimensional patch-based, multi-resolution pathway convolutional neural network architecture, DeepMedic. The model was trained to replicate physician segmentations using an ensemble of three networks trained with 5-fold cross-validation. The maximum SUV (SUV_max_), MTV, total lesion glycolysis (TLG), surface-area-to-volume ratio (SA/MTV), and a measure of disease spread (Dmax_patient_) were extracted from the model output. Pearson’s correlation coefficient and relative percent differences were calculated between automated and physician-extracted features.

**Results:**

Median Dice similarity coefficient of patient contours between automated and physician contours was 0.86 (IQR 0.78–0.91). Automated SUV_max_ values matched exactly the physician determined values in 81/100 cases, with Pearson’s correlation coefficient (*R*) of 0.95. Automated MTV was strongly correlated with physician MTV (*R* = 0.88), though it was slightly underestimated with a median (IQR) relative difference of − 4.3% (− 10.0–5.7%). Agreement of TLG was excellent (*R* = 0.94), with median (IQR) relative difference of − 0.4% (− 5.2–7.0%). Median relative percent differences were 6.8% (*R* = 0.91; IQR 1.6–4.3%) for SA/MTV, and 4.5% (*R* = 0.51; IQR − 7.5–40.9%) for Dmax_patient_, which was the most difficult feature to quantify automatically.

**Conclusions:**

An automated method using an ensemble of multi-resolution pathway 3D CNNs was able to quantify PET imaging features of lymphoma on baseline FDG PET/CT images with excellent agreement to reference physician PET segmentation. Automated methods with faster throughput for PET quantitation, such as MTV and TLG, show promise in more accessible clinical and research applications.

**Supplementary Information:**

The online version contains supplementary material available at 10.1186/s40658-020-00346-3.

## Introduction

Approximately 10–15% of pediatric cancers are malignant lymphomas, with about 40% of these lymphomas being Hodgkin lymphoma (HL) [1]. Treatment options for pediatric HL generally have favorable outcomes, with 5-, 10-, and 15-year survival rates of 95%, 93%, and 91%, respectively [2]. Pediatric patients, however, are uniquely vulnerable to therapeutic toxicities and their potential side effects later in life (e.g., infertility, secondary cancers). Several studies have shown therapies can be de-escalated in early responding pediatric HL patients, reducing the risk for long-term toxicities [3, 4]. Clinical trials have incorporated patient-specific risk stratification based upon interim therapy positron emission tomography (PET) response assessment, with the goal of overcoming resistance and reducing unnecessary therapy toxicity [5]. Current ^18^F-fluorodeoxyglucose (FDG) PET response assessment for both clinical and research studies uses a visual response assessment following a 5-point Deauville score [6]. However, quantitative PET metrics extracted from disease on baseline FDG PET/CT images have shown potential for accurate early risk stratification for both adult [7–12] and pediatric [13, 14] HL patients. These metrics most commonly include maximum uptake (SUV_max_); other metrics such as metabolic tumor volume (MTV) and total lesion glycolysis (TLG) are more involved technically.

Despite their clinical utility, the full potential of quantitative PET imaging metrics may not be reached in both adult and pediatric lymphoma due to the difficulty of delineating the entire lymphoma volume. Lymphoma can be highly heterogeneous in shape, size, and location. In order for physicians to extract quantitative PET information from disease, an analysis workflow can take up to 30–45 min per patient for difficult cases [15]. For comparison, feature extraction guided by automated methods can consistently reduce analysis times to less than 10 min per patient [15]. In addition, high rates of inter-observer variability in detection and interpretation are attributed to both the experience of the observer and to the extent of the patient’s disease [16].

Several methods have been developed that automatically detect and segment disease on FDG PET/CT images of adult lymphoma patients specifically [17–20] and in a large database including adult lung cancer and lymphoma patients [21]. Reporting in these previous studies has been limited to detection or segmentation performance (e.g., Dice similarity coefficients) or performance of classifiers. The impact of final lymphoma segmentation performance on subsequent quantitative PET feature extraction has not been assessed. In addition, automated quantification of prognostic PET metrics has not been assessed in pediatric lymphoma.

The purpose of this work was to develop a fully automated method for extraction of PET features for pediatric HL. The model was trained and tested using PET images of pediatric HL patients acquired at multiple centers as part of a multi-center clinical trial.

## Materials and methods

### Patient population

Patients included in this study were enrolled in a multi-center Children’s Oncology Group (COG) clinical trial, AHOD0831 high-risk pediatric HL phase 3 clinical trial (NCT01026220) using risk-adapted therapy in pediatric patients with high-risk HL [5]. Patients were located across North America and FDG PET/computed tomography (CT) images were acquired at a variety of imaging centers. All patients were under the age of 21 with high-risk (stage IIIB or IVB) HL. Baseline FDG PET/CT images were gathered and transferred from IROC-Rhode Island where they were permanently archived to the University of Wisconsin-Madison. One hundred of 166 patients enrolled on this clinical trial with good quality PET/CT images amenable for PET quantitative analysis were selected for retrospective analysis.

Using Mirada XD (Oxford, UK) imaging software, PET images for each patient were analyzed by one of two nuclear medicine physicians with experience and board certification in nuclear medicine (JK and IL). Large template regions of interest (ROIs) were placed around areas containing disease (nodal, spleen, and liver), excluding areas of osseous/bone marrow involvement and normal physiology such as the heart. Two thresholding segmentation methods were then applied (40% of tumor SUV_max_ and SUV > 2.5) within the template ROIs for segmentation of the lymphoma disease. Final segmentations, which were used for training and testing of the CNN, were generated by taking the union of the output of the two thresholding methods. This was done to ensure small, low-uptake lesions that can occur next to large, high-uptake lesions were not missed due to thresholding based on SUV_max_. Small, 1-voxel islands that can occur with thresholding algorithms were removed.

### Image pre-processing

PET and CT images were resampled to a cubic voxel size (2 × 2 × 2 mm) using linear resampling and normalized such that values inside the patient had a mean of 0 and a variance of 1. Labels were resampled to the same voxel size using nearest neighbor resampling. Patients were split randomly for 5-fold cross-validation (*N* = 20 patients per fold).

### Convolutional neural network approach

**Fig. 1:**
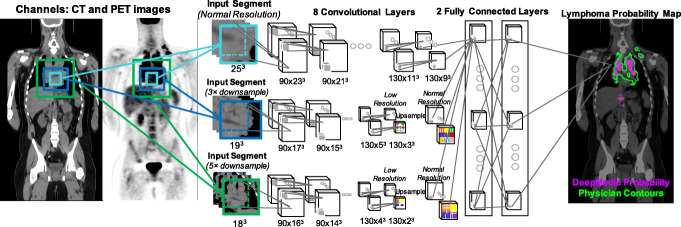
DeepMedic network design, adapted for lymph node detection. The number of features in all convolutional layers was 90, 90, 110, 110, 110, 110, 130, and 130. The number of features in the two fully-connected layers was 250. The input segment size of the downsampled pathways are set so that the feature maps prior to the final connected layer are of equivalent size, as in [22].

A 3D, multi-resolution pathway convolutional neural network (CNN), DeepMedic [22], was used (Fig. 1). The DeepMedic network has 8 convolutional layers (kernel size 3 × 3 × 3) for each resolution pathway, followed by two fully connected layers implemented as 1 × 1 × 1 convolutions and a final classification layer. Three resolution pathways were implemented, one at a normal image resolution, and two that down-sample the image by factors of 3 and 5 (thus increasing the receptive field by the same amount), which allows the network to consider context in addition to fine detail. Patches of size 25 × 25 × 25 voxels were extracted for training using class balancing of 50% of samples centered on a positive lymphoma voxel and 50% centered on a voxel not containing lymphoma.

DeepMedic [22] was trained for each fold of cross-validation. For each fold, 20 patients were in the training set, and the remaining 80 patients were split randomly such that 70 patients were used for training and 10 patients for validation. An ensemble CNN (3CNN) was created by training each model 3 times with different random initializations. Training was done on NVIDIA Tesla V100 GPUs.

The output of the CNN was further processed by applying the same thresholding scheme (union of SUV > 2.5 and SUV > 40% SUV_max_) within each of the contours generated by the 3 separately trained CNNs. The intersection of the 3 contours was taken as the final ensemble model output. As bone marrow activity was not considered in our model, any contours containing bone (CT Hounsfield Units > 150) were excluded from analysis.

### Statistical analysis

The performance of the final 3CNN model was assessed by measuring the sensitivity, positive predictive value (PPV), and Dice similarity coefficient (DSC) of a patient’s contours.

From final contours, quantitative imaging metrics were extracted, including SUV_max_, total body lymphoma MTV, and TLG. Two additional quantitative features were extracted for analysis: the ratio of tumor surface area to metabolic tumor volume (SA/MTV) and the distance between the two lesions that are farthest apart (Dmax_patient_), as both have been shown to be independent prognostic metrics in lymphoma [23, 24]. Automated feature extraction and physician-based feature extraction were compared using Pearson’s correlation coefficients, calculated for each extracted metric. In addition, relative percent differences were calculated and summarized with median and interquartile range values.

Subgroup analysis was performed to see if errors in segmentation and MTV estimation were influenced by certain characteristics of the subjects’ disease. Patient groups were dichotomized by median MTV, SA/MTV, and Dmax_patient_, and differences in DSC and MTV relative percent difference (RPD) were compared between subgroups. Wilcoxon rank sum tests were used to assess differences in DSC and absolute RPD of MTV.

## Results

A summary of the patient and scan characteristics is shown in Table 1. Patient ages ranged from 5 to 21 years with a median of 15.8 years, and 40/100 were female. Scans were acquired on nine different scanner models. Information on reconstruction settings for the images in this study is included in the Supplemental Material.

**Table 1:** Patient and scan characteristics

Sex (male/female/total)	60/40/100	
Patient age at enrollment, years Median (range)	15.8 (5.2, 21.4)	
Post-injection time, min Median (IQR)	73 (62, 82)	
Injected dose, (MBq) Median (IQR)	366.3 (270.1, 466.2)	
PET/CT scanners (*N* = number of scans)	Siemens Healthineers	Biograph (*N* = 5) Biograph HiRes (*N* = 12) Biograph TruPoint (*N* = 11)
	GE Medical Systems	Discover 690 (*N* = 5) Discovery LS (*N* = 13) Discovery RX (*N* = 3) Discovery ST/STE (*N* = 49) Advance (*N* = 1)
	Philips Healthcare	Gemini TF TOF 64 (*N* = 1)

**Fig. 2:**
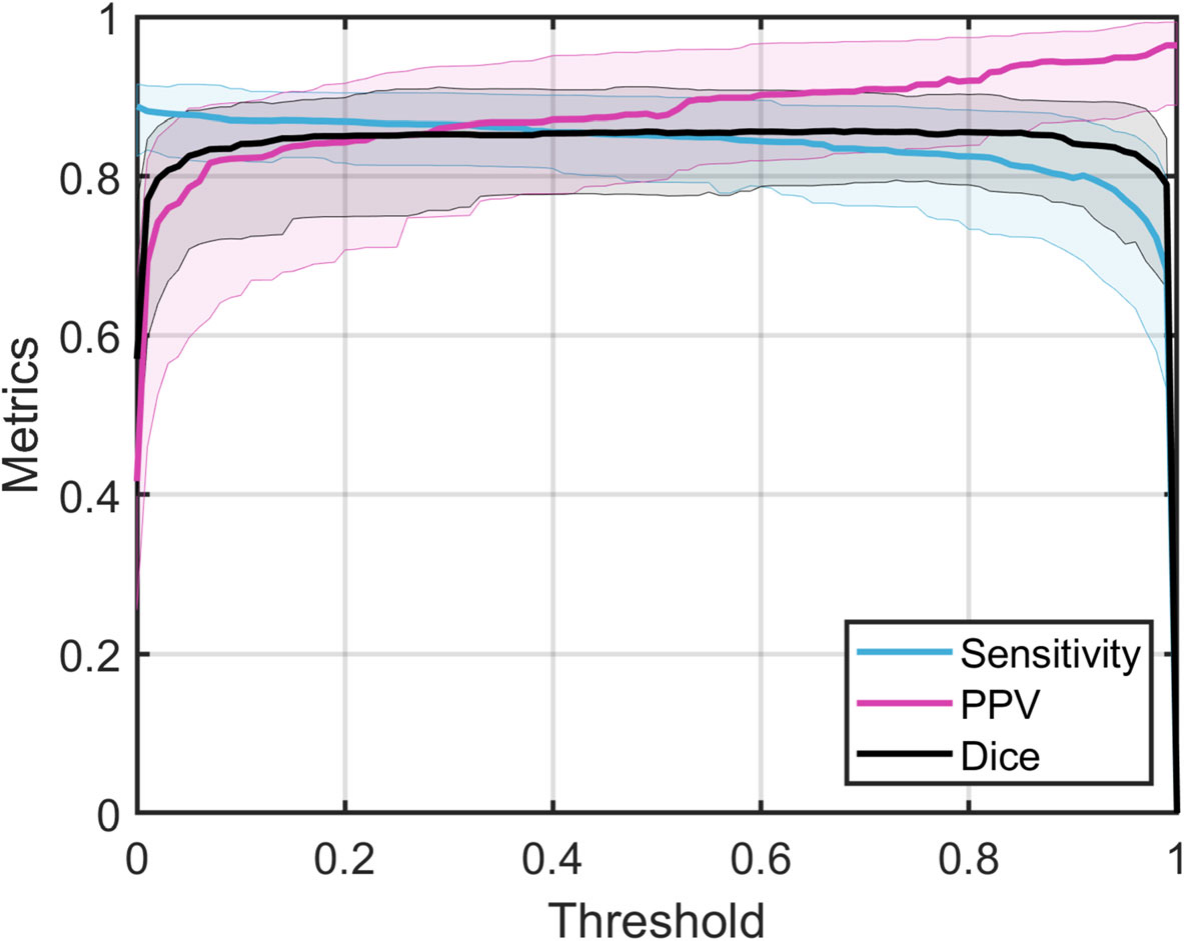
Segmentation performance as a function of baseline 3CNN probability threshold. Solid lines show median value across the 100 patients, shaded areas show interquartile range

Segmentation performance of the final model is shown in Fig. 2, plotted as a function of different cut points of the CNN’s probabilistic output. Using *p* = 0.5 as the cut point, median DSC was 0.86 (IQR 0.78–0.91). The median (IQR) for sensitivity and PPV were 0.85 (0.79–0.90) and 0.88 (0.80–0.96), respectively.

**Fig. 3:**
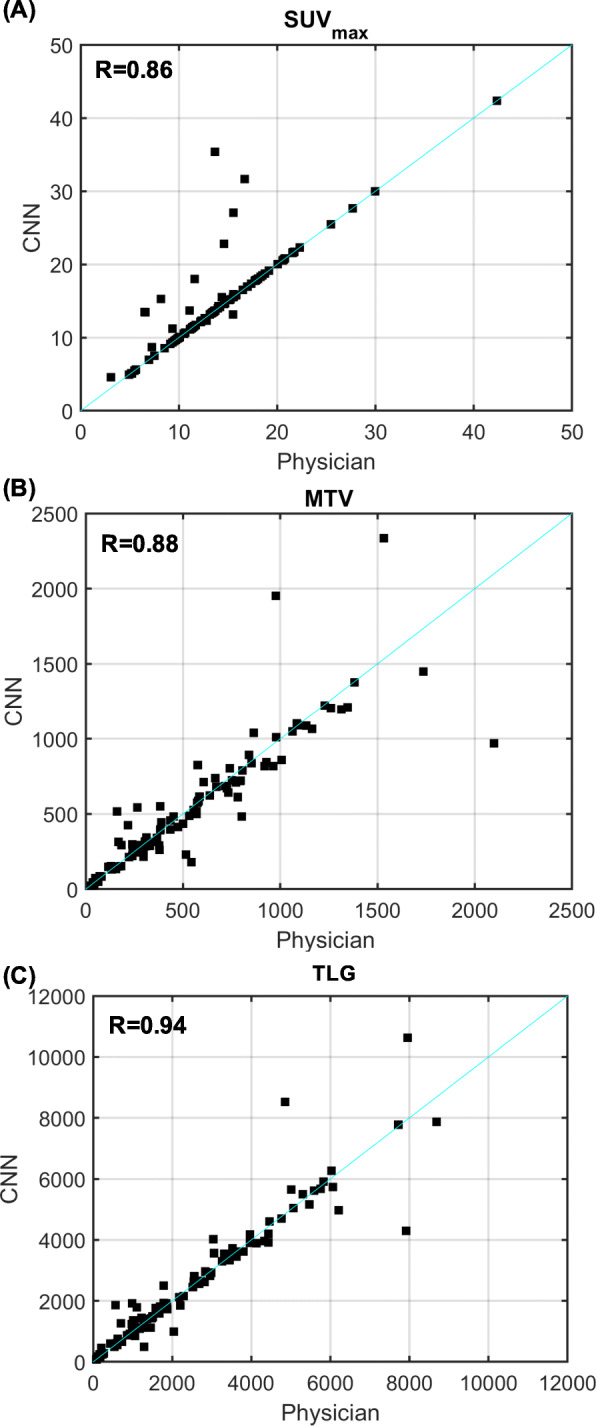
Comparison of physician-based and automatically extracted features: SUV_max_ (**a**), MTV (**b**), and TLG (**c**). Pearson correlation coefficients are shown in the top left corner of each plot, with unity lines shown in cyan

A comparison of PET metrics measured by physicians and by the model is shown in Fig. 3. SUV_max_ values matched exactly in 81/100 cases, with a Pearson’s correlation coefficient for SUV_max_ of *R* = 0.86. Automated MTV was strongly correlated with physician MTV (*R* = 0.88), though the model slightly underestimated MTV with a median (IQR) relative difference of − 4.2% (− 10–5.7%). Agreement of TLG was excellent (*R* = 0.94), with median (IQR) relative difference of − 0.4% (− 5.2–7.0%). Results were largely influenced by a handful of outlier patients (Fig. 3).

**Fig. 4:**
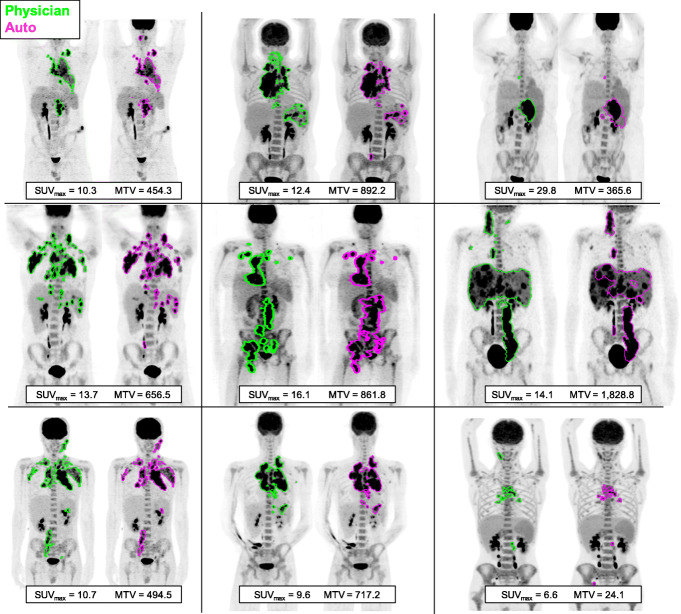
Example segmentation results of the physician contours (green) and automated method (magenta). Two examples of outlier are shown in the middle row: one for which MTV was largely overestimated (middle row, middle column) and one for which widespread liver disease was missed (middle row, right column). Units for SUV_max_ are in g/ml, and for MTV are in cm^3^, and values were extracted from physician contours

Examples of the model’s performance for 9 patients, including an outlier patient with MTV overestimation and one with MTV underestimation, are shown in Fig. 4. False-positive contours were most commonly located in the salivary glands, tonsils, and ureters.

**Fig. 5:**
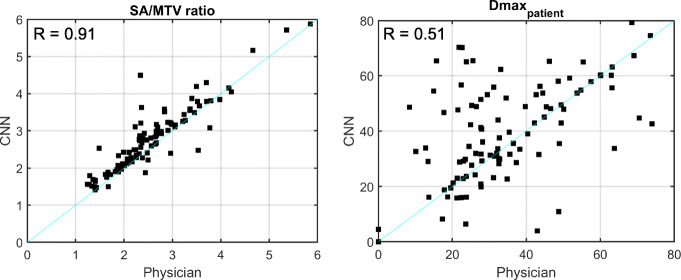
Comparison of SA/MTV (left) and dissemination of lesions (right) on baseline imaging between automated and physician-based contours. Pearson’s correlation coefficients are shown in top left of each plot

For SA/MTV and disease dissemination (Dmax_patient_), the agreement between the automated measurements and the physician measurements are shown in Fig. 5. Agreement for SA/MTV was much better than for Dmax_patient_, with median relative percent differences for SA/MTV of 6.8% (*R* = 0.91; IQR 1.6–14.3%) and for Dmax_patient_ of 4.5% (*R* = 0.51; IQR − 7.5–40.9%).

The impact of using an ensemble CNN approach as opposed to a single CNN is shown in Table 2. Performance of each of the three individual CNNs is shown and compared to the ensemble 3CNN performance. The ensemble approach resulted in a large improvement on SUV_max_ quantification, but did not have a large impact on the other quantitative metrics.

**Table 2:** Impact of using an ensemble of CNNs on quantification for the pediatric lymphoma population. Shown are results for SUV_max_, MTV, and TLG as summarized by Pearson’s correlation coefficient, *R*, and the relative percent difference when compared to physician measurements

	SUV_max_		MTV		TLG	
Method	***R***	Cases incorrect (of 100)	***R***	RPD Median [IQR]	***R***	RPD Median [IQR]
1st CNN initialization	0.73	24	0.89	− 3.5 [− 11.4, 4.2]	0.95	0.4 [− 5.2, 7.7]
2nd CNN initialization	0.74	25	0.87	− 4 [− 11.7, 3.9]	0.94	− 0.1 [− 5.1, 7]
3rd CNN initialization	0.59	24	0.89	− 3.8 [− 12.4, 5.3]	0.95	0 [− 6.7, 7.6]
Ensemble CNN	0.86	19	0.88	− 4.2 [− 10, 5.7]	0.94	− 0.4 [− 5.2, 7]

**Fig. 6:**
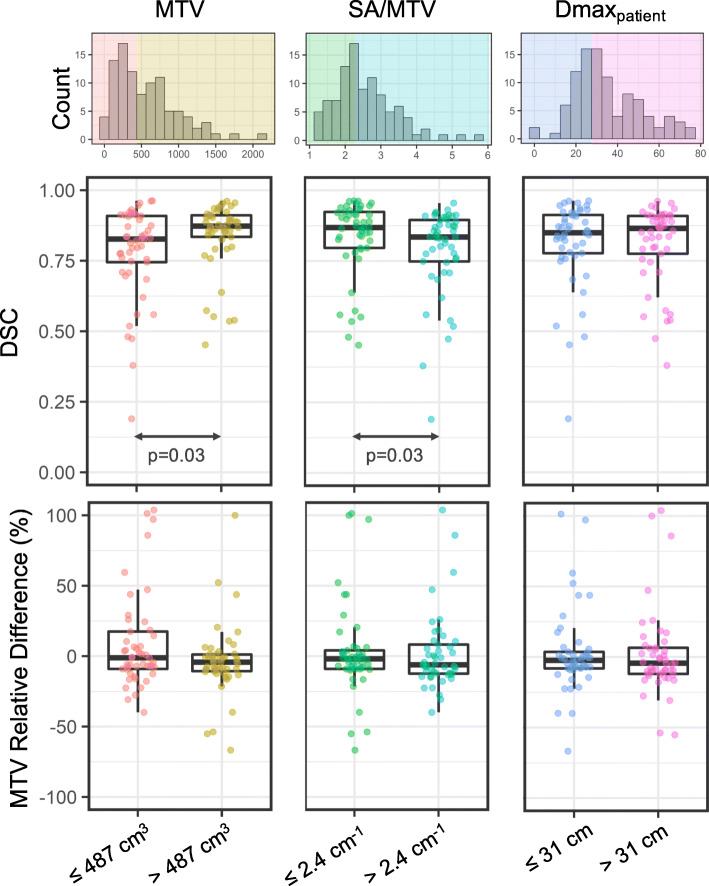
Subgroup analysis of the model’s segmentation and quantification performance. Disease was dichotomized by MTV (left), surface-area-to-volume ratio (SA/MTV, center), and dissemination of disease (Dmax_patient_, right) based on median values. Note plots are cropped, concealing an outlier at approximately 200% MTV RPD, for better visualization. Histograms in the top row show the spread of characteristics and are colored by median values

Results of the subgroup analysis are shown in Fig. 6. The accuracy of the automated MTV measurements was not significantly different between groups with high MTV and with low MTV. However, subjects with larger MTV had a significantly better DSC than those with smaller MTV (*p* = 0.03). In addition, lesions with smaller SA/MTV (i.e., more massive tumors) had significantly improved DSC compared to lesions with higher SA/MTV (i.e., more fragmented, smaller tumors, *p* = 0.03). A large spread in model performance was found across all subpopulations.

## Discussion

In this study, an ensemble of 3D convolutional neural networks was implemented for an automated assessment of 100 pediatric HL patients. Quantitative imaging features that have been shown to be prognostic on baseline PET images in HL (SUV_max_, MTV, TLG, and SA/MTV ratio) were able to be automatically extracted with excellent agreement with physician derived features. The implementation of an ensemble CNN showed significant improvements to using only a single CNN.

Our model-based measurement of volumetric quantitative PET metrics strongly agreed with physician-based quantification. This excellent performance was partially due to how physician segmentations were acquired. Because physicians applied PET SUV-based thresholding to label disease as opposed to manual contouring, we were able to apply those same thresholds within the CNN-detected contours, resulting in high Dice coefficients (median of 0.85, mean ± std of 0.81 ± 0.14). For comparison, a recent automated method for segmentation of lymphoma using 2D CNNs found mean ± std of DSC of 0.73 ± 0.06 when compared to manual physician contours in 80 adult patients [19]. Another method based on clustering of supervoxels found a DSC of 0.74 ± 0.08 in 48 adult patients based on physician contours using 41% SUV_max_ thresholding [25].

A metric describing the distance between the two lesions that are furthest apart, Dmax_patient_, was not easily replicated using automated contours in this study. This was primarily due to the model placing small false-positive regions in the salivary glands, tonsils, and ureters. This poor performance has implications for the use of a fully automated method without physician adjudication for staging of lymphoma, as staging considers whether disease is located on both sides of the diaphragm. As patients in this study were all high risk, stage III–IV, the majority of patients had disease on both sides of the diaphragm. It also illustrates that some imaging metrics are much more sensitive to full automation than others. For example, MTV is hardly affected when the model mistakenly contours very small benign regions, whereas these small false-positives can have a large impact on metrics like Dmax_patient_ or possibly staging.

The use of an ensemble CNN as opposed to an individual CNN was found to produce only moderate improvements for quantification of volume-based metrics (MTV and TLG). However, the ensemble model substantially improved measurements of SUV_max_. This was because the ensemble CNN reduced false-positives by ensuring all three CNNs positively identified each region. As the majority of false-positives were located in areas of high FDG uptake (ureter, salivary gland, tonsil), the reduction of false-positives had a significant impact on SUV_max_ quantification. Because the false-positives were typically small, the ensemble had only a minor impact on the other PET imaging metrics.

Consistent performance of MTV quantification was found across different subgroups of patients. However, significantly better performance in DSC was achieved in larger lesions (MTV > 487 cm^3^) and lesions with a low surface-area-to-volume ratio (SA/MTV < 2.4 cm^−1^). These significant differences in DSC across MTV are not surprising, as in general a high DSC value is easier to achieve in larger volumes. In addition, disease with a low SA/MTV are larger, more massive lesions, and are easier to contour with a high DSC value compared to smaller, more fragmented disease. More patients are needed to ensure this trend remains across a wider variety of disease types.

The main limitation of this study is that ground-truth contours were obtained from only a single physician using PET SUV-based thresholding techniques. This prevents an analysis of interphysician variability in labeling, although this interphysician variability is expected to be somewhat low given that PET SUV-based thresholding was used to define tumor boundaries. The use of thresholding as opposed to manual segmentation results in better repeatability across observers, but its use remains controversial due to its many limitations. This study was limited to high-risk stage IIIB and IVB pediatric HL patients. Similar results are expected for adult lymphoma populations with high disease burden [20]; however, it is unknown how this approach would perform in patients with a low disease burden. Lastly, a large number of PET/CT scanners from various institutions were used in this study, many of which were older scanner models. Thus, while the model is expected to better generalize than models that are trained using data from a single scanner or institution, it is unclear how accurate the model would perform on images acquired with new scanner models without the inclusion of images acquired on new scanner models during training.

## Conclusion

A fully automated, 3D CNN-based method of lymphoma identification and quantitation in PET/CT images showed good agreement with physician labels. Overall, these techniques show promise in standardizing and improving lymphoma patient care, while also expanding the potential of quantitative PET imaging in lymphoma.

## Supplementary Information

**Additional file 1.**

## Data Availability

Data is not available due to ethical concerns.

## Abbreviations

COG: Children’s Oncology Group
CNN: Convolutional neural networks
CT: Computed tomography
DSC: Dice similarity coefficient
HL: Hodgkin lymphoma
MTV: Metabolic tumor volume
PET: Positron emission tomography
ROI: Region of interest
SUV: Standardized uptake value
TLG: Total lesion glycolysis
