# Supplementary material for: Automated quantification of baseline imaging PET metrics on FDG PET/CT images of pediatric Hodgkin lymphoma patients
Source: EJNMMI Phys. 2020 Dec 14;7:76. doi: 10.1186/s40658-020-00346-3 (PMC7736382; doi:10.1186/s40658-020-00346-3)
Supplement: Supplementary file 1 — Additional file 1. [file 40658_2020_346_MOESM1_ESM.pdf]

Supplemental Material

Table 1: List of reconstruction parameters for each scanner used for image acquisition in this study.

**Scanner**	**Number of patients**	**Reconstruction methods**	**Voxel Size** [XY, Z] (mm)	**2D/3D**
GE Discovery 690	5	3D OSEM + TOF<br>3D OSEM	[3.65, 3.27]<br>[2.86, 3.27]	3D
GE Discovery LS	13	OSEM	[3.91, 4.25]<br>[4.30, 4.25]	2D
GE Discovery STE	49	3D IR<br>OSEM	[1.37, 3.27]<br>[3.91, 3.27]<br>[4.69, 3.27]<br>[5.47, 3.27]	2D<br>3D
GE Discovery RX	3	OSEM	[3.91, 3.27]<br>[4.69, 3.27]<br>[5.47, 3.27]	3D
GE Advance	1	*	[4.30, 4.25]	2D
Philips GEMINI TF TOF 64	1	BLOB-OS-TF	[4.00, 4.00]	3D
Siemens Biograph	5	OSEM<br>OSEM + PSF	[5.20, 2.43]<br>[5.31, 3.38]<br>[4.07, 4.00]	3D
Siemens HiRes	12	OSEM	[4.06, 2.00]	3D
Siemens TruePoint	11	OSEM<br>OSEM + PSF	[4.07, 5.00]<br>[4.07, 4.00]<br>[2.19, 5.00]<br>[2.78, 5.00]	3D

*values not available due to anonymization
OSEM: ordered subsets expectation maximization. TOF: time-of-flight. PSF: point spread function modelling. IR: iterative reconstruction. BLOB-OS: spherically symmetric basis function ordered subset algorithm
